# Three-Component Coupling Reactions of Arynes for the Synthesis of Benzofurans and Coumarins

**DOI:** 10.3390/molecules19010863

**Published:** 2014-01-13

**Authors:** Eito Yoshioka, Shigeru Kohtani, Hideto Miyabe

**Affiliations:** School of Pharmacy, Hyogo University of Health Sciences, 1-3-6, Minatojima, Chuo-ku, Kobe 650-8530, Japan

**Keywords:** arynes, multi-component reaction, domino reaction, heterocycles, synthesis

## Abstract

The domino three-component coupling reaction of arynes with DMF and active methylenes or methines was studied as a highly efficient method for preparing heterocycles. Coumarin derivative **5** was formed when diethyl malonate (**2**) or α-bromomalonate (**3**) were used as a C2-unit. In contrast, dihydrobenzofurans **7a** and **7b** were obtained by using α-chloroenolates generated from α-chloromalonates **4a** and **4b** and Et_2_Zn. The benzofuran **15a** could be obtained by using ethyl iodoacetate (**14**) as a C1-unit. The one-pot conversion of dihydrobenzofurans **7a**, **7b** and **8a** into benzofurans **15a** and **15b** was also studied. The direct synthesis of benzofuran **15b** was achieved by using the active methine **18** having ketone and ester groups.

## 1. Introduction

Synthetic strategies involving domino or cascade process offer the advantage of multiple carbon-carbon and/or carbon-heteroatom bond formations in a single operation [1]. In recent years, the domino reactions using arynes continue to attract much interest [2,3,4,5,6,7,8,9,10,11,12,13,14,15,16,17,18,19,20,21], since arynes are highly reactive species for constructing the multi-substituted arenes with structural diversity and complexity [22,23].

The recent aryne-based chemistry has achieved some remarkable success in the transition metal-catalyzed reactions [2,3,4,5,6,7,8,9,10], the transition metal-free reactions and other transformations [11,12,13,14,15,16,17,18,19,20,21]. These advances have shown that the insertion of arynes into various element-element σ-bonds can be achieved even under the transition metal-free conditions. We have been interested in developing the corresponding π-bond insertion [24,25,26,27,28,29,30,31]. Recently, we reported the efficient insertion into the C=O bond of formamides [32,33], which was successfully applied into the domino process trapping the transient intermediates with nucleophiles [34,35,36]. In this paper, we describe in detail our approach to prepare coumarin, dihydrobenzofuran and benzofuran derivatives via the three-component coupling process starting from arynes generated from *ortho*-(trimethylsilyl)aryl triflates.

## 2. Results and Discussion

### 2.1. New Approach for the Domino Three-Component Coupling Process

**Scheme 1:**
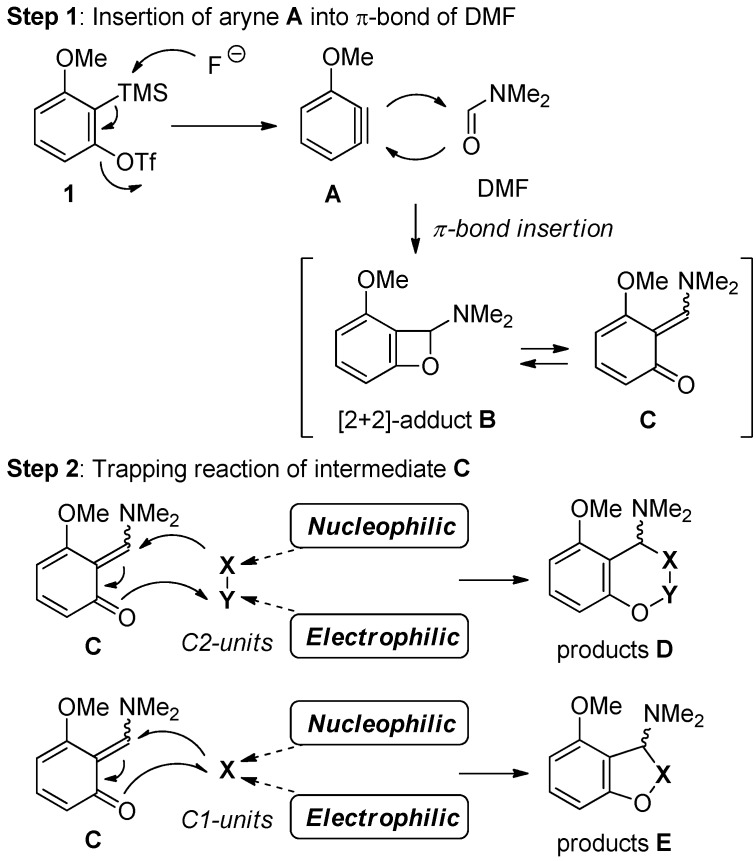
Three-component coupling reaction.

The goal of our study on aryne chemistry is to develop the highly efficient domino reactions for preparing heterocycles. Therefore, we have designed a new approach involving two steps which are induced by the high reactivity related to the strain energy of aryne **A** and the four-membered intermeditate **B** (Scheme 1) [37]. 

The insertion of a highly strained aryne **A**, generated *in situ* from *ortho*-(trimethylsilyl)aryl triflate **1** and the fluoride ion [38], into the C=O of *N*,*N*-dimethylformamide (DMF) gives the moderately strained [2+2] adduct benzoxetene **B**, which would undergo isomerized into *ortho*-quinone methide **C** (Step 1). The sequential transformation can be achieved by the initial addition of nucleophiles to the transient intermediate **C** and the subsequent trapping process with electrophiles (Step 2). When nucleophile and electrophile belong to the same molecule as shown in Scheme 1, the use of C2-units (**X**–**Y**) leads to the products **D** and the use of C1-units (**X**) leads to the products **E**.

**Scheme 2:**
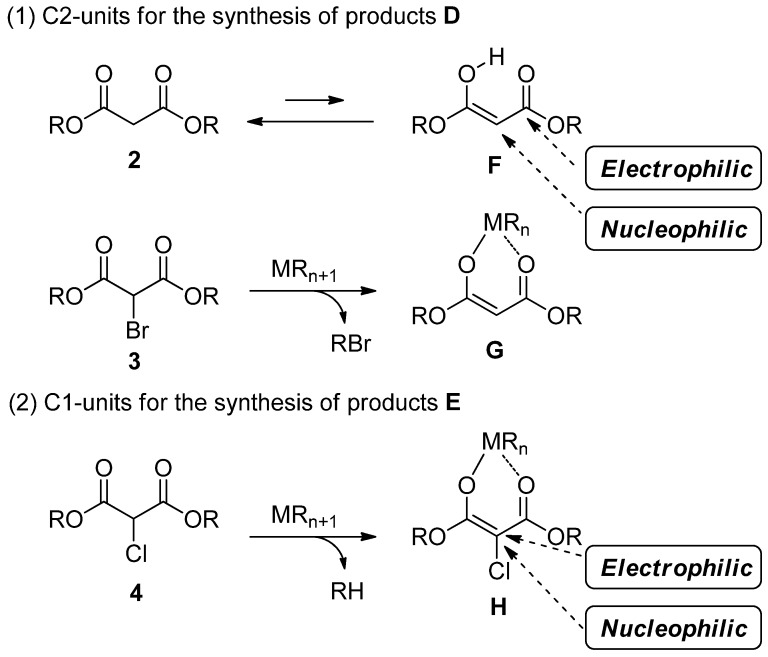
Substrates for trapping the intermediate **C**.

For the synthesis of products **D** such as coumarin derivatives, we used enol **F** and enolate **G** having both nucleophilic and electrophilic sites, which were derived from malonate **2** and α-bromomalonate **3**, respectively (Scheme 2). For the synthesis of products **E** such as dihydrobenzofurans and benzofurans, α-chloroenolate **H** having a nucleophilic and electrophilic carbon atom, derived from α-chloromalonate **4**, was employed for trapping the unstable intermediate **C**.

### 2.2. The Synthesis of Coumarin Derivative

In organic synthesis, DMF can react as either an electrophilic or nucleophilic agent [39,40]. At first, we examined the reaction of 3-methoxy-2-(trimethylsilyl)phenyl triflate (**1**) as an aryne precursor with DMF and diethyl malonate (**2**) as a C2-unit (Table 1). It is well known that the active methylenes such as diethyl malonate (**2**) have an excellent reactivity toward arynes giving the σ-bond insertion products [41,42,43,44,45]. To suppress the competitive insertion of aryne into the C–C σ-bond of **2**, DMF was employed as a solvent. We were gratified to observe the sufficient reactivity of active methylene **2** toward intermediate **B** in the absence of base. The effect of fluoride ion sources was studied. In the presence of CsF, treatment of triflate **1** with **2** in DMF at room temperature predominantly gave the desired coumarin **5** in 65% yield, accompanied by a trace amount of salicylaldehyde derivative **6** (entry 1). The replacement of CsF with anhydrous TBAF led to an increase in the chemical yield to give **5** in 86% yield (entry 2). In contrast, no reaction was observed when KF was employed (entry 3).

**Table 1:** Reaction of aryne precursor **1** with DMF and **2** ^a^. 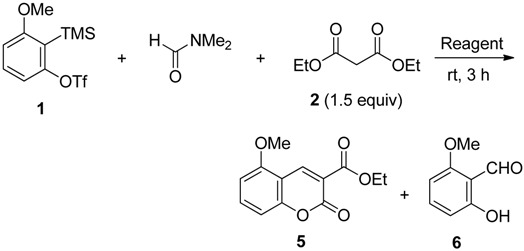

Entry	Reagent (3.0 equiv)	Product (% yield) ^b^
1	CsF	5 (65), 6 (trace)
2	TBAF	5 (86)
3	KF	NR ^c^

^a^ Reactions were carried out with **1** (1.0 equiv), **2** (1.5 equiv), and reagent (3.0 equiv) in DMF (0.1 M solution of **1**). ^b^ Isolated yield. ^c^ No reaction; Triflate **1** was recovered in 93% yield.

**Scheme 3:**
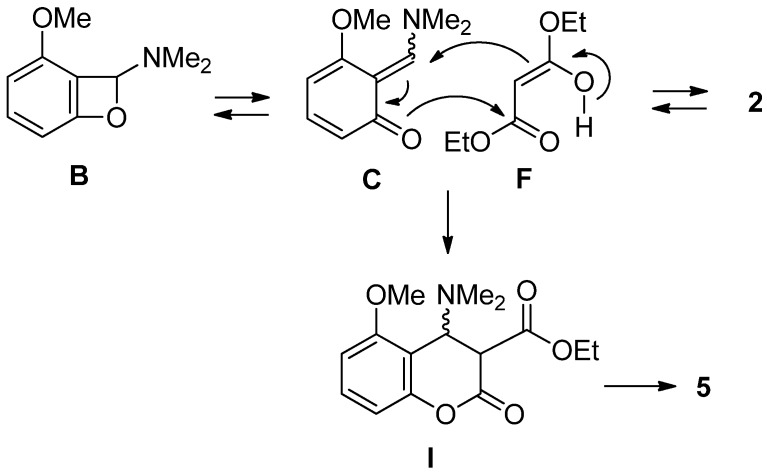
Reaction pathway.

This domino transformation involves the trapping reaction of the unstable intermediate **C** with enol **F** giving the intermediate **I** (Scheme 3). The coumarin **5** was formed via the elimination of a dimethylamino group from the intermediate **I**.

Further investigations using α-bromomalonate **3** and organometallic reagents such as Et_2_Zn or Me_3_Al were performed (Table 2). In the presence of Et_2_Zn, we initially allowed triflate **1** to react with **3** in DMF at room temperature for 12 h (entry 1). The desired coumarin **5** was obtained in 11% yield, accompanied by the recovered triflate **1** in 64%. Although the replacement of CsF with anhydrous TBAF led to an increase in the chemical yield, the new formation of dihydrobenzofuran **7a** was observed (entry 2). The reaction did not take place when KF was employed (entry 3). Therefore, Me_3_Al was next employed (entries 4 and 5). In the presence of CsF, treatment of **1** with **3** in DMF predominantly gave the desired product **5** in 34% yield (entry 4). Improvement in the chemical yield of **5** was observed when anhydrous TBAF was used (entry 5). The chemical yield increased into 85%. In this transformation, a suitable combination of α-bromomalonate **3** and Me_3_Al led to the efficient generation of the debrominated metal enolate **G**, which reacted with intermediate **C** to give coumarin **5**.

**Table 2:** Reaction of aryne precursor **1** with DMF and **3** ^a^. 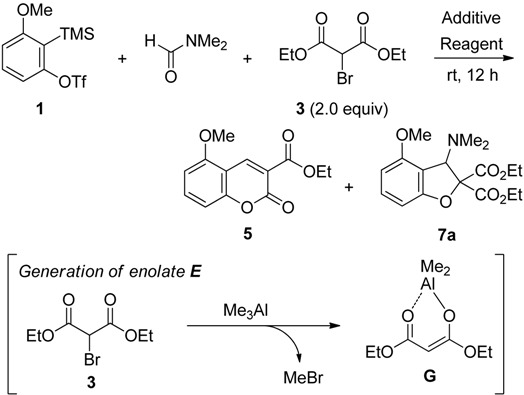

Entry	Reagent (5.0 equiv)	Additive (2.0 equiv)	Product (% yield) ^b^
1	CsF	Et_2_Zn	**5** (11) ^c^
2	TBAF	Et_2_Zn	**5** (41), **7a** (23)
3	KF	Et_2_Zn	NR ^d^
4	CsF	Me_3_Al	**5** (34) ^e^
5	TBAF	Me_3_Al	**5** (85)

^a^ Reactions were carried out with **1** (1.0 equiv), **3** (1.5 equiv), reagent (5.0 equiv), and additive (2.0 equiv) in DMF (0.1 M solution of **1**). ^b^ Isolated yield. ^c^ Triflate **1** was recovered in 64% yield. ^d^ No reaction; Triflate **1** was recovered in 98% yield. ^e^ Triflate **1** was recovered in 12% yield.

### 2.3. The Synthesis of Dihydrobenzofurans

**Scheme 4:**
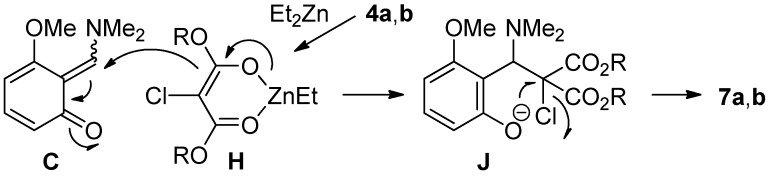
Generation of enolates and reaction pathway.

We next investigated the domino reaction for the synthesis of dihydrobenzofurans (Table 3). The key issue of this transformation is the efficient generation of α-halogenated enolate as a C1-unit. However, as mentioned above, the debromination took place when α-bromomalonate **3** and organometallic reagents were employed. In remarked contrast to α-bromomalonate **3**, we found that the use of α-chloromalonates **4a**,**b** and Et_2_Zn led to the generation of desired α-halogenated enolates **H** (Scheme 4). Thus, a combination of α-chloromalonates **4a**,**b** and Et_2_Zn was checked under the different reaction conditions for the synthesis of dihydrobenzofurans.

**Table 3:** Reaction of aryne precursor **1** with DMF and **4a**,**b**. 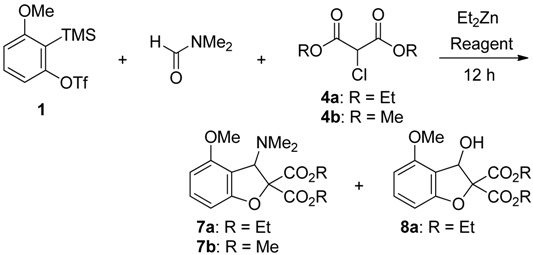

Entry	Methine	Reagent	*T*	Product (% yield) ^a^
1 ^b^	4a	TBAF	rt	**7a** (21), **8a** (64)
2 ^b^	4a	TBAF	−40 °C to rt	**7a** (66), **8a** (24)
3 ^b^	4a	CsF	−40 °C to rt	**7a** (63)
4 ^c^	4a	CsF	−40 °C to rt	**7a** (86)
5 ^b^	4a	KF	rt	NR ^d^
6 ^b^	4b	CsF	−40 °C to rt	**7b** (70)
7 ^c^	4b	CsF	−40 °C to rt	**7b** (89)

^a^ Isolated yield. ^b^ Reactions were carried out with **1** (1.0 equiv), **4a**,**b** (2.0 equiv), reagent (5.0 equiv), and Et_2_Zn (2.0 equiv) in DMF (0.1 M solution of **1**). ^c^ Reactions were carried out with **1** (1.2 equiv), **4a**,**b** (1.0 equiv), CsF (6.0 equiv), and Et_2_Zn (1.0 equiv) in DMF (0.1 M solution of **1**). ^d^ No reaction; Triflate **1** was recovered in 95% yield.

In the presence of anhydrous TBAF, treatment of triflate **1** with **4a** in DMF at room temperature gave the desired product **7a** in 21% yield, accompanied by 64% yield of undesired dihydrobenzofuran **8a** (entry 1). The undesired dihydrobenzofuran **8a** having a hydroxy group would be formed as a result of hydrolysis of intermediates **B** or **C** with contamining water. The isolated yield of **7a** increased to 66% yield by changing the reaction temperature (entry 2). The formation of undesired product **8a** was not observed when CsF was employed (entries 3 and 4). In particular, improvement in the chemical yield of **7a** was observed, when 1.2 equivalents of triflate **1** was reacted with 1.0 equivalent of **4a** in DMF (entry 4). Similar trend was observed in the reaction using α-chloromalonate **4b** (entries 6 and 7). In the presence of CsF and Et_2_Zn, treatment of triflate **1** (1.2 equiv) with **4b** (1.0 equiv) in DMF at −40 °C to room temperature for 12 h gave the desired dihydrobenzofuran **7b** in 89% yield (entry 7).

In this transformation, α-chloroenolates **H** are effectively generated from α-chloromalonates **4a**,**b** and Et_2_Zn (Scheme 4). These α-halogenated enolates **H** work as not only a nucleophile to attack to the intermediate **C** but also an electrophile to trap intramolecularly the intermediate anion **J** to give the desired dihydrobenzofurans **7a**,**b**.

**Scheme 5:**
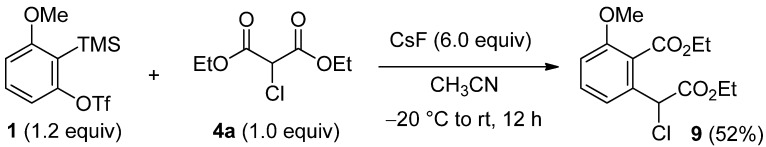
Reaction of **1** with **4a**.

The reactivity of α-chloromalonate **4a** toward arynes was also investigated (Scheme 5). In the presence of CsF, the direct reaction of triflate **1** with **4a** was carried out in CH_3_CN without DMF. As expected, the σ-bond insertion product **9** was obtained in 52% yield.

**Scheme 6:**
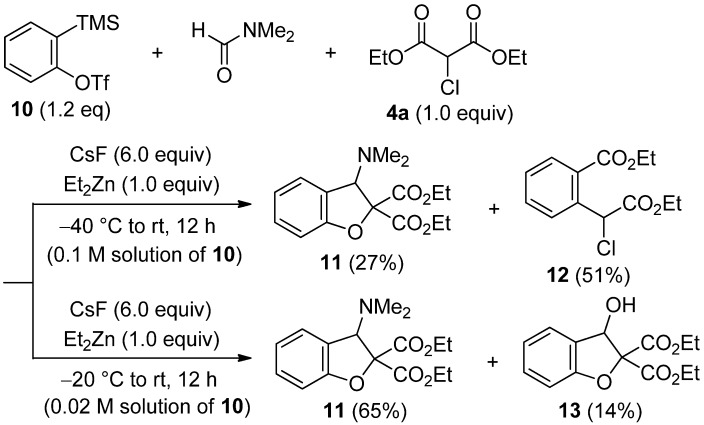
Reaction of **10** with DMF and **4a**.

As mentioned above, the competitive insertion of aryne into the C–C σ-bond of **4a** was not observed in the domino three-component coupling reaction of bulky triflate **1**. Decreasing the steric hindrance around the triple bond of aryne induced the direct insertion of aryne into α-chloromalonate **4a**. When sterically less hindered triflate **10** was employ as an aryne precursor, the σ-bond insertion product **12** was obtained in 51% yield (Scheme 6). To suppress the competitive insertion of aryne into **4a**, the concentration was evaluted. Under the high diluted concentration (0.02 M solution of **10** in DMF), the σ-bond insertion was mostly suppressed to afford the desired dihydrobenzofuran **11** in 65% yield, accompanied by 14% yield of dihydrobenzofuran **13** having a hydroxy group.

### 2.4. The Synthesis of Benzofurans

With these results in mind, the synthesis of benzofurans was next studied (Table 4). At first, ethyl iodoacetate **14** was employed as a C1-unit. The reaction of triflate **1** with **14** was run in DMF in the presence of 3.0 equivalents of TBAF (entry 1). However, the simple *O*-alkylated product **16** was formed in 28% yield, accompanied by salicylaldehyde derivative **6** in 45% yield. The similar trend was observed when CsF was used (entry 2). The reaction temperature had an impact on the chemical transformation (entry 3). The desired benzofuran **15a** was obtained in 40% yield, when reaction was run at 100 °C. The use of Et_2_Zn or Me_3_Al as additive was not effective for this reaction (entries 4 and 5).

**Table 4:** Reaction of aryne precursor **1** with DMF and **14** ^a^. 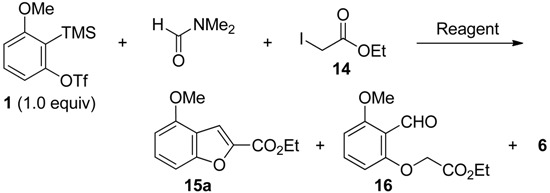

Entry	Reagent (equiv)	Ethyl iodoacetate	*T* (°C)	Time (h)	Product (% yield) ^b^
1	TBAF (3.0)	1.5 equiv	rt	12	**16** (28), **6** (45)
2	CsF (3.0)	1.5 equiv	rt	12	**16** (44), **6** (34)
3	CsF (5.0)	2.0 equiv	100	3	**15a** (40), 16 (trace), **6** (11)
4 ^c^	CsF (5.0)	2.0 equiv	rt	24	Complex mixture ^d^
5 ^e^	CsF (5.0)	2.0 equiv	rt	24	NR ^f^

^a^ Reactions were carried out with **1** (1.0 equiv), **14** (1.5 or 2.0 equiv), and reagent (3.0 or 5.0 equiv) in DMF (0.1 M solution of **1**). ^b^ Isolated yield. ^c^ Reaction was carried out in the presence of Et_2_Zn (2.0 equiv). ^d^ Triflate **1** was recovered in 36% yield. ^e^ Reaction was carried out in the presence of Me_3_Al (2.0 equiv). ^f^ No reaction; Triflate **1** was recovered in 79% yield.

**Scheme 7:**
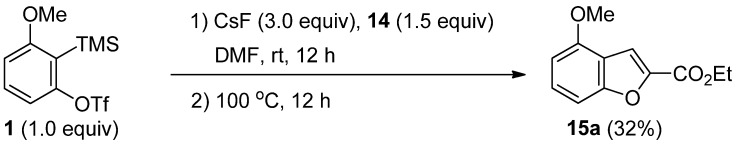
Conversion of **16** into **15a**.

To understand the reaction pathway, the formation of benzofuran **15a** from the simple *O*-alkylated product **16** was studied (Scheme 7). As expected, benzofuran **15a** was obtained in 32% yield, after being stirred at room temperature for 12 h followed by heated at 100 °C for 12 h. Thus, benzofuran **15a** could be obtained from *O*-alkylated product **16**.

**Scheme 8:**
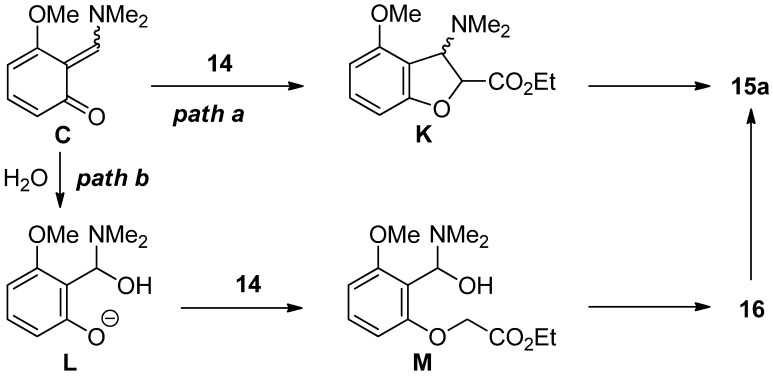
Two reaction pathways.

For the formation of benzofuran **15a**, two possible reaction pathways are shown in Scheme 8. As a direct pathway, benzofuran **15a** is assumed to be obtained from *ortho*-quinone methide **C** and **14** via intermediate **K** (*path a*). Another pathway is the formation of benzofuran **15a** from the simple *O*-alkylated product **16** via intermediates **L** and **M** (*path b*).

**Scheme 9:**
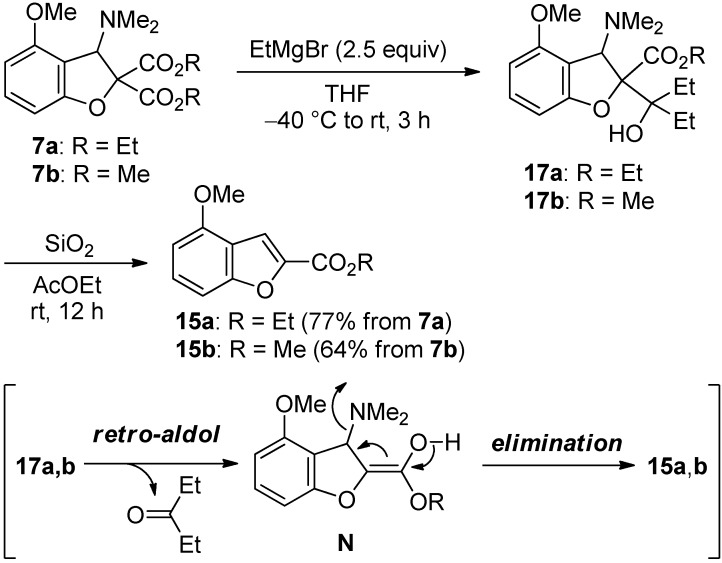
Conversion of **7a**,**b** into **15a**,**b**.

As an alternative approach for synthesis of benzofurans, we tried to establish the conversion of dihydrobenzofurans **7a** and **7b** into benzofurans **15a** and **15b** (Scheme 9). When dihydrobenzofuran **7a** was treated with 2.5 equivalents of EtMgBr followed by SiO_2_, the disered benzofuran **15a** was obtained in 77% yield without the isolation of adduct **17a**. Similarly, benzofuran **15b** was formed form dihydrobenzofuran **7b**. These transformations would proceed via the retro-aldol type reaction of adducts **17a** and **17b** followed by the elimination of a dimethylamino group.

**Scheme 10:**
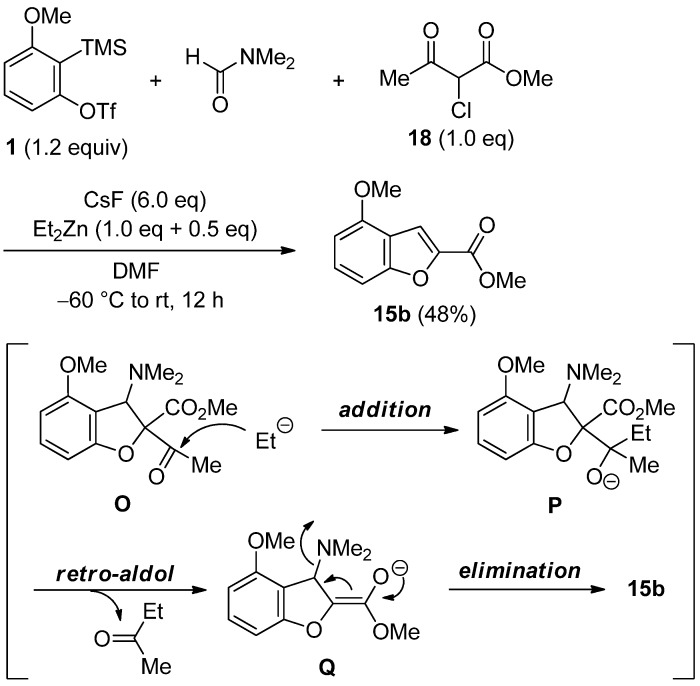
Direct one-pot synthesis of benzofuran **15b**.

Next, we directed our attention into the direct one-pot synthesis of benzofuran **15b** (Scheme 10). For this purpose, the active methine **18** having ketone and ester groups was used, since ketone moiety would selectively react with Et_2_Zn, leading to the retro-aldol type process. In the presence of CsF, triflate **1** and methine **18** in DMF were treated with Et_2_Zn (1.0 equiv + 0.5 equiv) at −60 °C to room temperature for 12 h. As expected, the desired benzofuran **15b** having an ester group was directly generated via the addition of an ethyl anion to a ketone group of dihydrobenzofuran **O**, the retro-aldol type reaction of intermediate **P** and the elimination of a dimethylamino group of anion **Q**.

**Scheme 11:**
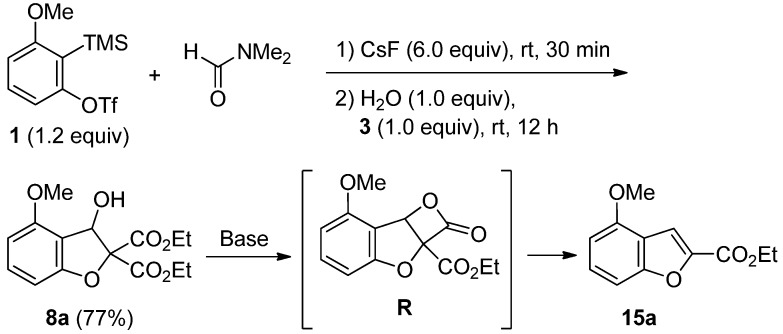
Preparation of **8a** and transformation of **8a** into **15a**.

Finally, we investigated the transformation of dihydrobenzofuran **8a** having a hydroxy group into benzofuran **15a** (Scheme 11) [46]. As a starting substrate, the preparation of dihydrobenzofuran **8a** was initially studied. When the domino reaction of triflate **1** with α-bromomalonate **3** and DMF was carried out in the presence of water (1.0 equiv), the desired dihydrobenzofuran **8a** was obtained in 77% yield instead of dihydrobenzofuran **7a** having a dimethylamino group. For the synthesis of benzofuran **15a**, we next allowed dihydrobenzofuran **8a** to react with several bases (Table 5). Treatment of dihydrobenzofuran **8a** with 1.0 equivalent of NaH in DMF at room temperature gave the desired benzofuran **15a** in 83% yield (entry 1). Probably, this transformation proceeds via the decarboxylation of cyclic intermediate **R**. In contrast, benzofuran **15a** was not obtained when LiHMDS was employed in THF at −40 °C (entry 2). Interestingly, the replacement of LiHMDS with NaHMDS led to the formation of **15a** (entry 3). The isolated yield of **15a** dramatically increased to 96% yield by replacing NaHMDS with KHMDS (entry 4).

**Table 5:** Synthesis of benzofuran **15a** ^a^.

Entry	Base (1.0 equiv)	Solvent	*T* (°C)	Time (h)	Yield (%) ^b^
1	NaH	DMF	rt	16	83
2	LiHMDS	THF	−40	88	NR ^c^
3	NaHMDS	THF	−40	88	11
4	KHMDS	THF	−40	16	96

^a^ Reactions were carried out with **8a** (1.0 equiv) and base (1.0 equiv). ^b^ Isolated yield. ^c^ No reaction; Starting substrate **8a** was recovered in 84% yield.

## 3. Experimental

### 3.1. General

Melting points were taken on a Yanaco MP-J3 and are uncorrected. Infrared spectra were measured on a JASCO FT/IR-4100. ^1^H-NMR spectra were measured on a JEOL ECX-400 PSK (400 MHz) or Varian NMRS 600 (600 MHz). ^13^C-NMR spectra were measured on a JEOL ECX-400 PSK (101 MHz) or Varian NMRS 600 (151 MHz) with CDCl_3_ as an internal standard (77.0 ppm). High resolution mass spectra were obtained by use of a Hitachi M-4100 GC/MS spectrometer or Thermo Fisher Scientific Exactive LC/MS spectrometer. For silica gel column chromatography, SiliCycle Inc. SiliaFlash F60 was used. The anhydrous TBAF was prepared from TBAF·3H_2_O by heating the hydrate at 40 °C for 6 h, at 60 °C for 12 h, at 80 °C for 6 h, and then at 120 °C for 12 h under reduced pressure [47]. The prepared anhydrous TBAF was used as a solution by addition of appropriate solvent such as DMF.

### 3.2. Procedure for the Synthesis of Coumarin Derivative **5** using Malonate **2**

To a solution of 3-methoxy-2-(trimethylsilyl)phenyl triflate (**1**, 105 µL, 0.40 mmol) and diethyl malonate (**2**, 91 µL, 0.60 mmol) in DMF (3.4 mL) was added a solution of anhydrous TBAF (314 mg, 1.2 mmol) in DMF (0.6 mL) under argon atmosphere at room temperature. After being stirred at room temperature for 3 h, silica gel (1.0 g) was added to the reaction mixture, and then it was concentrated under reduced pressure. Purification of the residue by flash silica gel column chromatography (AcOEt/hexane = 1:8–1:0 with 2% CH_2_Cl_2_) afforded coumarin derivative **5** (85 mg, 86%).

*5-Methoxy-2-oxo-2H-1-benzopyran-3-carboxylic acid, Ethyl ester* (**5**). Pale yellow crystals. mp 132.5–133.5 °C (CH_2_Cl_2_-*iso*-Pr_2_O). IR (KBr) 2981, 1764, 1704, 1609, 1478 cm^−1^. ^1^H-NMR (CDCl_3_) δ 8.90 (1H, s), 7.55 (1H, t, *J* = 8.0 Hz), 6.93 (1H, br d, *J* = 8.0 Hz), 6.73 (1H, dd, *J* = 8.0, 0.5 Hz), 4.41 (2H, q, *J* = 7.0 Hz), 3.97 (3H, s), 1.41 (3H, t, *J* = 7.0 Hz). ^13^C-NMR (CDCl_3_) δ 163.3, 157.4, 156.9, 156.2, 144.1, 135.2, 116.0, 109.0, 108.9, 105.2, 61.8, 56.2, 14.3. HRMS (ESI) calcd for C_13_H_13_O_3_ (M+H^+^): 249.0763. Found: 249.0754. Elemental analysis (%) calcd for C_13_H_12_O_5_: C, 62.90; H, 4.87. Found: C, 62.69; H, 5.01.

### 3.3. Procedure for the Synthesis of Coumarin Derivative **5** using α-Bromomalonate **3**

To a solution of diethyl α-bromomalonate (**3**, 68 µL, 0.40 mmol) in DMF (1.5 mL) was added Me_3_Al (1.08 M in hexane, 370 µL, 0.40 mmol) under argon atmosphere at room temperature. After being stirred at the same temperature for 5 min, 3-methoxy-2-(trimethylsilyl)phenyl triflate (**1**, 53 µL, 0.20 mmol) and TBAF (264 mg, 1.00 mmol) in DMF (0.5 mL) were added to the reaction mixture. After being stirred at the same temperature for 12 h, the reaction mixture was diluted with saturated NaHCO_3_ and then extracted with CH_2_Cl_2_. The organic phase was dried over Na_2_SO_4_ and concentrated at reduced pressure. Purification of the residue by flash silica gel column chromatography (AcOEt/hexane = 1:20–1:1 with 2% CH_2_Cl_2_) afforded coumarin derivative **5** (42 mg, 85%).

### 3.4. Typical Procedure for the Synthesis of Dihydrobenzofurans

To a suspension of CsF (183 mg, 1.20 mmol) in DMF (2.0 mL) was added Et_2_Zn (1.0 M in toluene, 200 µL, 0.20 mmol) under argon atmosphere at −40 °C. After being stirred at the same temperature for 5 min, diethyl α-chloromalonate **4a** (32 µL, 0.20 mmol) and 3-methoxy-2-(trimethylsilyl)phenyl triflate (**1**, 63 µL, 0.24 mmol) were added to the reaction mixture. After being stirred at −40 °C to room temperature for 12 h, silica gel (0.5 g) was added to the reaction mixture, and then it was concentrated under reduced pressure. Purification of the residue by flash silica gel column chromatography (EtOAc/hexane = 1:20–1:4) afforded dihydrobenzofuran **7a** (58.0 mg, 86%). Under similar reaction conditions, dihydrobenzofurans **7b** and **11** were synthesized. Products **8a**, **12** and **13** were also formed.

*3-(Dimethylamino)-4-methoxy-2,2(3H)-benzofurandicarboxylic acid, 2,2-Diethyl ester* (**7a**). Colorless oil. IR (KBr) 2982, 1741, 1601, 1492, 1460 cm^−1^. ^1^H-NMR (CDCl_3_) δ 7.18 (1H, t, *J* = 8.0 Hz), 6.61 (1H, d, *J* = 8.0 Hz), 6.50 (1H, d, *J* = 8.0 Hz), 5.17 (1H, s), 4.40–4.12 (4H, m), 3.83 (3H, s), 2.22 (6H, br s), 1.30 (3H, t, *J* = 7.0 Hz), 1.25 (3H, t, *J* = 7.0 Hz). ^13^C-NMR (CDCl_3_) δ 167.3, 165.7, 159.4, 157.5, 130.9, 111.3, 103.8 (2C), 94.6, 69.8, 62.3, 61.7, 55.2, 43.0, 14.1, 13.9. HRMS (ESI^+^) calcd for C_17_H_24_NO_6_ (M+H^+^): 338.1598, Found: 338.1593.

*3-(Dimethylamino)-4-methoxy-2,2(3H-benzofurandicarboxylic acid, 2,2-Dimethyl ester* (**7b**). Colorless oil. IR (KBr) 2952, 2839, 1773, 1746, 1601, 1458 cm^−1^. ^1^H-NMR (CDCl_3_) δ 7.19 (1H, t, *J* = 8.2 Hz), 6.61 (1H, d, *J* = 8.2 Hz), 6.51 (1H, d, *J* = 8.2 Hz), 5.17 (1H, s), 3.84 (3H, s), 3.83 (3H, s), 3.77 (3H, s), 2.23 (6H, br s). ^13^C-NMR (CDCl_3_) δ 167.8, 166.1, 159.3, 157.5, 131.0, 111.1, 103.9 (2C), 94.8, 70.1, 55.2, 53.4, 52.7, 43.0 (br). HRMS (ESI^+^) calcd for C_15_H_19_NO_6_Na (M+Na^+^): 332.1105, Found: 332.1145.

*3-Hydroxy-4-methoxy-2,2(3H)-benzofurandicarboxylic acid, 2,2-Diethyl ester* (**8a**). Colorless oil. IR (KBr) 3504, 2983, 1741, 1606, 1494, 1465 cm^−1^. ^1^H-NMR (CDCl_3_) δ 7.24 (1H, t, *J* = 8.0 Hz), 6.63 (1H, d, *J* = 8.0 Hz), 6.50 (1H, d, *J* = 8.0 Hz), 6.01 (1H, br d, *J* = 4.5 Hz), 4.38–4.18 (4H, m), 3.85 (3H, s), 2.68 (1H, br s), 1.31 (3H, t, *J* = 7.0 Hz), 1.28 (3H, t, *J* = 7.0 Hz). ^13^C-NMR (CDCl_3_) δ 166.2, 165.1, 159.8, 157.2, 132.4, 113.2, 104.6, 103.7, 93.2, 74.5, 62.7, 62.6, 55.6, 14.0, 13.9. HRMS (EI^+^) calcd for C_15_H_18_O_7_Na (M+Na^+^): 333.0945, Found: 333.0942.

*3-(Dimethylamino)-2,2(3H)-benzofurandicarboxylic acid, 2,2-Diethyl ester* (**11**). Colorless oil. IR (KBr) 2982, 2940, 1769, 1742, 1598, 1472, 1462 cm^−1^. ^1^H-NMR (CDCl_3_) δ 7.27–7.23 (2H, m), 7.00–6.95 (2H, m), 5.08 (1H, s), 4.38–4.12 (4H, m), 2.18 (6H, br s), 1.31 (3H, t, *J* = 7.1 Hz), 1.25 (3H, t, *J* = 7.1 Hz). ^13^C-NMR (CDCl_3_) δ 167.2, 165.8, 157.7, 129.9, 125.8, 123.2, 121.6, 110.9, 93.2, 70.7, 62.5, 61.9, 42.4 (br), 14.1, 13.9. HRMS (ESI^+^) calcd for C_16_H_22_NO_5_ (M+H^+^): 308.1492, Found: 308.1586.

*α-Chloro-2-(ethoxycarbonyl)benzeneacetic acid, Ethyl ester* (**12**). Colorless oil. IR (KBr) 2983, 2935, 1749, 1716, 1601, 1578, 1466, 1448 cm^−1^. ^1^H-NMR (CDCl_3_) δ 8.00 (1H, dd, *J* = 7.8, 1.4 Hz), 7.80 (1H, dd, *J* = 7.8, 1.4 Hz), 7.59 (1H, td, *J* = 7.6, 1.4 Hz), 7.43 (1H, td, *J* = 7.6, 1.4 Hz), 6.52 (1H, s), 4.38 (2H, q, *J* = 7.2 Hz), 4.28–4.19 (2H, m), 1.40 (3H, t, *J* = 7.2 Hz), 1.26 (3H, t, *J* = 7.3 Hz). ^13^C-NMR (CDCl_3_) δ 168.6, 166.6, 137.1, 132.7, 130.7, 129.7, 128.9, 128.7, 62.4, 61.5, 55.9, 14.2, 13.9. HRMS (ESI^+^) calcd for C_13_H_15_^35^ClO_4_Na (M+Na^+^): 293.0551, Found: 293.0548; HRMS (ESI^+^) calcd for C_13_H_15_^37^ClO_4_Na (M+Na^+^): 295.0522, Found: 295.0517.

*3-Hydroxy-2,2(3H)-benzofurandicarboxylic acid, 2,2-Diethyl ester* (**13**). IR (KBr) 3491, 2984, 1741, 1601, 1477, 1468 cm^−1^. ^1^H-NMR (CDCl_3_) δ 7.41 (1H, br d, *J* = 7.3 Hz), 7.31 (br td, *J* = 7.8, 1.4 Hz), 7.04–7.00 (2H, m), 5.91 (1H, br d, *J* = 4.6 Hz), 4.38–4.21 (4H, m), 2.74 (1H, br s), 1.32 (3H, t, *J* = 7.1 Hz), 1.29 (3H, t, *J* = 7.1 Hz). ^13^C-NMR (CDCl_3_) δ 166.4, 165.3, 158.4, 131.2, 125.8, 125.7, 122.5, 111.0, 92.8, 76.4, 62.8, 62.7, 14.1, 13.9. HRMS (ESI^+^) calcd for C_14_H_16_O_6_Na (M+Na^+^): 303.0839, Found: 303.0843.

### 3.5. Procedure for the Insertion into α-Chloromalonate **4a**

To a suspension of CsF (183 mg, 1.2 mmol) in MeCN (2.0 mL) were added diethyl α-chloromalonate (**4a**, 32 µL, 0.20 mmol) and 3-methoxy-2-(trimethylsilyl)phenyl triflate (**1**, 63 µL, 0.24 mmol) under argon atmosphere at −20 °C. After being stirred at −20 °C to room temperature for 12 h, the reaction mixture was diluted with saturated NaHCO_3_ and then extracted with AcOEt. The organic phase was dried over Na_2_SO_4_ and concentrated at reduced pressure. Purification of the residue by PTLC (AcOEt/hexane =1:2) afforded the product **9** (31 mg, 52%).

*α-Chloro-[2-(ethoxycarbonyl)-3-methoxy]benzeneacetic acid, Ethyl ester* (**9**). IR (KBr) 2983, 1752, 1736, 1589, 1472, 1442 cm^−1^. ^1^H-NMR (CDCl_3_) δ 7.40 (1H, t, *J* = 8.2 Hz), 7.25 (1H, br d, *J* = 8.5 Hz), 6.93 (1H, br d, *J* = 8.5 Hz), 5.50 (1H, s), 4.42 (2H, q, *J* = 7.1 Hz), 4.27–4.15 (2H, m), 3.83 (3H, s), 1.39 (3H, t, *J* = 7.1 Hz), 1.24 (3H, t, *J* = 7.1 Hz). ^13^C-NMR (CDCl_3_) δ 167.8, 166.5, 156.6, 134.6, 131.2, 123.2, 120.4, 111.9, 62.6, 61.7, 56.1, 55.6, 14.1, 13.9. HRMS (ESI^+^) calcd for C_14_H_17_^35^ClO_5_Na (M+Na^+^): 323.0657, Found: 323.0642; HRMS (ESI^+^)calcd for C_14_H_17_^37^ClO_5_Na (M+Na^+^): 325.0633, Found: 325.0613.

### 3.6. Procedure for the Synthesis of Benzofuran **15a**

To a suspension of CsF (304 mg, 2.0 mmol) in DMF (4.0 mL) were added 3-methoxy-2-(trimethylsilyl)phenyl triflate (**1**, 105 µL, 0.40 mmol) and ethyl iodoacetate **14** (95 µL, 0.80 mmol) under argon atmosphere at 100 °C. After being stirred at the same temperature for 12 h, the reaction mixture was diluted with saturated NaHCO_3_ and then extracted with CH_2_Cl_2_. The organic phase was dried over Na_2_SO_4_ and concentrated at reduced pressure. Purification of the residue by flash silica gel column chromatography (EtOAc/hexane = 1:20–1:4) afforded the product **15a** (35 mg, 40%). Product **16** was also formed.

*4-Methoxy-2-benzofurancarboxylic acid, Ethyl ester* (**15a**). Colorless oil. IR (KBr) 2981, 1726, 1609, 1570, 1500 cm^−1^. ^1^H-NMR (CDCl_3_) δ 7.62 (1H, d, *J* = 1.0 Hz), 7.35 (1H, t, *J* = 8.2 Hz), 7.18 (1H, d, *J* = 8.2 Hz), 6.67 (1H, d, *J* = 8.2 Hz), 4.43 (2H, q, *J* = 7.1 Hz), 3.94 (3H, s), 1.41 (3H, t, *J* = 7.1 Hz). ^13^C-NMR (CDCl_3_) δ 159.5, 156.9, 154.6, 144.4, 128.5, 117.8, 111.6, 105.1, 103.5, 61.4, 55.6, 14.3. HRMS (ESI^+^) calcd for C_12_H_13_O_4_ (M+H^+^): 221.0808, Found: 221.0806.

*2-(2-Formyl-3-methoxyphenoxy)acetic acid, Ethyl ester* (**16**). IR (KBr) 2981, 1756, 1689, 1597, 1474 cm^−1^. ^1^H-NMR (CDCl_3_) δ 10.57 (1H, s), 7.42 (1H, t, *J* = 8.5 Hz), 6.63 (1H, d, *J* = 8.5 Hz), 6.44 (1H, d, *J* = 8.5 Hz), 4.72 (2H, s), 4.26 (2H, q, *J* = 7.0 Hz), 3.90 (3H, s), 1.28 (3H, t, *J* = 7.0 Hz). ^13^C-NMR (CDCl_3_) δ 189.2, 168.2, 161.9, 160.6, 135.6, 114.9, 105.1, 104.8, 66.0, 61.5, 56.1, 14.1. HRMS (ESI^+^) calcd for C_12_H_15_O_5_ (M+H^+^): 239.0920. Found: 239.0912.

### 3.7. Typical Procedure for Conversion of Dihydrobenzofurans into Benzofurans

To a solution of **7a** (40.0 mg, 0.12 mmol) in THF (2.4 mL) was added EtMgBr (1.0 M in THF, 300 µL, 0.30 mmol) under argon atmosphere at −40 °C. After being stirred at −40 °C to room temperature for 3 h, the reaction mixture was diluted with saturated NH_4_Cl and then extracted with AcOEt. The organic phase was dried over Na_2_SO_4_ and concentrated at reduced pressure to give quantitatively the crude adduct **17a**, which was used for next reaction without further purification. To a solution of **17a** (35.2 mg, 0.10 mmol) in AcOEt (1.0 mL) was added silica gel (0.50 g) under the atmosphere at room temperature. After being stirred for 12 h, the reaction mixture was concentrated under reduced pressure. Purification of the residue by flash silica gel column chromatography (EtOAc/hexane = 1:10–1:3) afforded the product **15a** (16.9 mg, 77%).

*4-Methoxy-2-benzofurancarboxylic acid, Methyl ester* (**15b**). Colorless oil. IR (KBr) 2952, 2844, 1733, 1609, 1500 cm^−1^. ^1^H-NMR (CDCl_3_) δ 7.63 (1H, br s), 7.37 (1H, t, *J* = 8.2 Hz), 7.19 (1H, d, *J* = 8.2 Hz), 6.68 (1H, d, *J* = 8.2 Hz), 3.97 (3H, s), 3.96 (3H, s). ^13^C-NMR (CDCl_3_) δ 160.0, 156.9, 154.7, 144.1, 128.7, 117.9, 111.9, 105.1, 103.5, 66.7, 52.3. HRMS (ESI^+^) calcd for C_11_H_10_O_4_Na (M+Na^+^): 229.0471, Found: 229.0472.

### 3.8. Procedure for Direct Synthesis of Benzofuran **15b**

To a suspension of CsF (183 mg, 1.20 mmol) in DMF (2.0 mL) was added Et_2_Zn (1.0 M in toluene, 200 µL, 0.20 mmol) under argon atmosphere at −60 °C. After being stirred at the same temperature for 5 min, methyl 2-chloroacetoacetate (**18**, 24 µL, 0.20 mmol) and 3-methoxy-2-(trimethylsilyl)phenyl triflate (**1**, 63 µL, 0.24 mmol) were added to the reaction mixture. After being stirred at −60 °C to room temperature for 12 h, Et_2_Zn (1.0 M in toluene, 100 µL, 0.10 mmol) was added to the reaction mixture. After being stirred for 3 h, silica gel (0.5 g) was added to the reaction mixture, and then it was concentrated under reduced pressure. Purification of the residue by flash silica gel column chromatography (EtOAc/hexane = 1:20–1:4) afforded the product **15b** (19.9 mg, 48%).

### 3.9. Procedure for Transformation of Dihydrobenzofuran **8a** into Benzofuran **15a**

To a solution of dihydrobenzofuran **8a** (50 mg, 0.16 mmol) in THF (3.2 mL) was added KHMDS (0.50 M in toluene, 320 µL, 0.16 mmol) under argon atmosphere at −40 °C. After being stirred at the same temperature for 12 h, the reaction mixture was diluted with saturated NaHCO_3_ and then extracted with CH_2_Cl_2_. The organic phase was dried over Na_2_SO_4_ and concentrated at reduced pressure. Purification of the residue by PTLC (EtOAc/hexane = 1:4 with 2% CH_2_Cl_2_) afforded benzofuran **15a** (33 mg, 96%).

## 4. Conclusions

We have demonstrated that the domino three-component coupling reaction of arynes with DMF and active methylenes or methines gave various heterocycles such as coumarin derivatives, dihydrobenzofurans and benzofurans.
